# Using Speech Data From Interactions With a Voice Assistant to Predict the Risk of Future Accidents for Older Drivers: Prospective Cohort Study

**DOI:** 10.2196/27667

**Published:** 2021-04-08

**Authors:** Yasunori Yamada, Kaoru Shinkawa, Masatomo Kobayashi, Hironobu Takagi, Miyuki Nemoto, Kiyotaka Nemoto, Tetsuaki Arai

**Affiliations:** 1 IBM Research Tokyo Japan; 2 Department of Psychiatry University of Tsukuba Hospital Ibaraki Japan; 3 Department of Psychiatry Faculty of Medicine University of Tsukuba Ibaraki Japan

**Keywords:** cognitive impairment, smart speaker, speech analysis, accident, prevention, older adults, prediction, risk, assistant

## Abstract

**Background:**

With the rapid growth of the older adult population worldwide, car accidents involving this population group have become an increasingly serious problem. Cognitive impairment, which is assessed using neuropsychological tests, has been reported as a risk factor for being involved in car accidents; however, it remains unclear whether this risk can be predicted using daily behavior data.

**Objective:**

The objective of this study was to investigate whether speech data that can be collected in everyday life can be used to predict the risk of an older driver being involved in a car accident.

**Methods:**

At baseline, we collected (1) speech data during interactions with a voice assistant and (2) cognitive assessment data—neuropsychological tests (Mini-Mental State Examination, revised Wechsler immediate and delayed logical memory, Frontal Assessment Battery, trail making test-parts A and B, and Clock Drawing Test), Geriatric Depression Scale, magnetic resonance imaging, and demographics (age, sex, education)—from older adults. Approximately one-and-a-half years later, we followed up to collect information about their driving experiences (with respect to car accidents) using a questionnaire. We investigated the association between speech data and future accident risk using statistical analysis and machine learning models.

**Results:**

We found that older drivers (n=60) with accident or near-accident experiences had statistically discernible differences in speech features that suggest cognitive impairment such as reduced speech rate (*P*=.048) and increased response time (*P*=.040). Moreover, the model that used speech features could predict future accident or near-accident experiences with 81.7% accuracy, which was 6.7% higher than that using cognitive assessment data, and could achieve up to 88.3% accuracy when the model used both types of data.

**Conclusions:**

Our study provides the first empirical results that suggest analysis of speech data recorded during interactions with voice assistants could help predict future accident risk for older drivers by capturing subtle impairments in cognitive function.

## Introduction

As the world's older adult population increases, car accidents involving older adults have become an increasingly serious social problem. While it has been reported that older drivers have an increased risk of car accident involvement per unit distance travelled [1-4], they also showed a substantially higher rate of serious injury than that of middle-age car drivers [5,6]. Even in normal aging, there is a decline in many cognitive abilities related to driving, and this cognitive decline is known to be one of the risk factors for older adults being involved in car accidents [7-9]. Associating cognitive assessment scores with either self-reported car accidents, crash records, or on-road driving measures has been investigated to identify predictors of driving safety (in previous empirical studies [7]). In particular, cognitive abilities such as visual attention, short-term memory, and executive functions (evaluated with neuropsychological tests) were consistently shown to have associations with driving safety [7,10-12]. In this respect, if cognitive impairments relevant to driving safety in older adults can be inferred solely from behavior data in everyday situations in a passive way, this would be beneficial for accident prevention.

Speech in daily life can be used a potential data sources for determining cognitive impairments related to driving safety. Speech involves multiple interacting cognitive abilities including attention, memory, and executive functions [13,14]. Many empirical studies have used speech data to identify cognitive impairments resulting from aging and diseases such as Alzheimer disease [15-18] and characterized speech changes related to cognitive impairments by extracting linguistic and paralinguistic features from speech data [19-25]. For example, difficulties with word finding and word retrieving have been quantified by tallying pronoun frequency and pause durations [19,20,24,26-28]. A reduction in speech expressiveness has also been quantified by measuring lexical diversity and speech rate [19,23,29-31]. Using a combination of these features, previous studies [19-25,29] have succeeded in differentiating individuals with cognitive impairments from healthy controls. Although no study has investigated the relationship between speech data and driving safety, it is reasonable to explore the possibility that speech data could be used for inferring ability to drive safely from changes in cognitive functioning in older drivers.

At the same time, there is growing interest in using speech data that can be collected in everyday situations for applications in health care owing to the popularity of voice-based interaction systems such as voice assistants in smart speakers and smartphones [32-34]. One approach is to provide various types of voice-based tests via a smart-speaker platform. For example, previous studies [35,36] have used mobile apps for collecting speech responses to neuropsychological tasks such as verbal fluency and picture description tasks; they showed accurate classification rates in detecting patients with Alzheimer disease [35] and dementia [36]. Another approach is to analyze health-related insights from speech data collected during daily voice-based interactions. For example, vocal characteristics in speech data during typical tasks on smart speakers appeared to be associated with neuropsychological test scores [37], while linguistic features extracted from phone conversation data were significant indicators for differentiating patients with Alzheimer disease from older adults with normal cognition [38]. This approach, focusing on speech data that can be collected in everyday situations, would increase opportunities for frequent assessment by facilitating passive and unobtrusive monitoring.

In this study, we aimed to investigate the relationship between speech data and future driving experiences related to car accidents in healthy older adults by collecting speech data during interactions with a voice assistant with simulated tasks on smart speakers and smartphones. We hypothesized that these speech data could be used for predicting accident risk for older drivers.

## Methods

### Participants

We recruited healthy older adults aged 60 years or older through recruiting agencies and advertisements in the local community in Ibaraki, Japan. All examinations were conducted in Japanese. Older adults met the inclusion criteria if they were in good physical and mental health and had no serious diseases, disabilities, mental illness (eg, major depression, bipolar disorder, and schizophrenia), or neurodegenerative diseases (eg, Parkinson disease and dementia). This study was conducted with the approval of the University of Tsukuba Hospital Ethics Committee (H29-065). All participants provided written consent after the procedures of the study had been fully explained.

A total of 71 older individuals participated in the cognitive assessments and speech data collection (women: 38/71, 53.5%; age: range 61-80 years, mean 71.1, SD 4.9). Of the original 71 participants, 60 consented to the follow-up study about their driving experiences (women: 33/60, 55.0%; age: range 61-80 years, mean 70.8, SD 5.1; Table 1). They were contacted again approximately one-and-a-half years after the speech data collection (mean 17.3 months, SD 2.7) and answered a questionnaire on their driving experiences within the past year. The questionnaire included free-form questions about accidents and near accidents; *near accidents* were described as infractions and any other incidents while driving that they deemed to be dangerous regardless of severity and culpability.

**Table 1:** Demographic and assessment data for study participants.

Variable			Total (N=60)		Individuals without accident or near-accident experiences (n=34)		Individuals with accident or near-accident experiences (n=26)		*P* value
Age (years), mean (SD)			70.8 (5.1)		70.5 (4.9)		71.3 (5.3)		.45
Education (years), mean (SD)			13.7 (2.2)		13.7 (2.2)		13.6 (2.1)		.93
**Sex, n (%)**									.53
	Men	27 (45)		17 (50)		10 (38)			
	Women	33 (55)		17 (50)		16 (62)			
Mini-Mental State Examination^a^, mean (SD)			27.6 (1.8)		27.4 (1.8)		27.9 (1.8)		.28
LM IA^b^, mean (SD)			9.6 (3.8)		9.1 (3.7)		10.2 (4.0)		.43
LM IIA^c^, mean (SD)			7.5 (3.6)		7.3 (3.7)		7.6 (3.6)		.74
Frontal Assessment Battery^d^, mean (SD)			13.7 (2.7)		13.4 (2.7)		14.2 (2.7)		.45
Trail making test-part A (seconds), mean (SD)			33.2 (9.8)		33.6 (9.7)		32.6 (10.1)		.72
Trail making test-part B (seconds), mean (SD)			89.5 (49.7)		95.7 (60.9)		81.3 (28.2)		.71
Clock Drawing Test^e^, mean (SD)			6.7 (0.8)		6.7 (0.7)		6.7 (1.0)		.36
Geriatric Depression Scale^f^, mean (SD)			2.9 (2.4)		2.8 (2.4)		3.1 (2.4)		.62
Severity scores for atrophy in medial temporal structures, mean (SD)			0.9 (0.6)		0.8 (0.4)		0.9 (0.7)		.86

^a^The total possible score ranges from 0 to 30.

^b^LM IA: immediate recall of the logical memory-story A of the Wechsler memory scale-revised for episodic memory; the total possible score ranges from 0 to 25.

^c^LM IIA: delayed recall of the logical memory-story A of the Wechsler memory scale-revised for episodic memory; the total possible score ranges from 0 to 25.

^d^The total possible score ranges from 0 to 18.

^e^The total possible score ranges from 0 to 7.

^f^The total possible score ranges from 0 to 15.

### Cognitive Assessments

Cognitive assessments and examinations were those typically used for the diagnosis of dementia and comprised 12 variables: age, sex, education, 7 neuropsychological test scores (Mini-Mental State Examination for global cognition; immediate and delayed recall of the logical memory-story A of the Wechsler memory scale-revised for episodic memory; the Frontal Assessment Battery for executive function; the trail making test-part A and B for executive function and attention; and the clock drawing test for visuospatial function), and 2 clinical scores (Geriatric Depression Scale and the severity of medial temporal lobe atrophy). The severity of medial temporal lobe atrophy was evaluated using structural magnetic resonance imaging (MRI) scans—1.5 T, T1-weighted images and a 3D gradient-echo sequence—with the following parameters: sagittal orientation with 1.2-mm thick sections; time repetition/time echo: 2400/3.52 milliseconds; flip angle: 8°; field of view: 192×192. We expressed the severity of medial temporal lobe atrophy as a *Z* score relative to cognitively healthy adults by using a standalone, voxel-based specific regional analysis system for Alzheimer disease [39]. Two psychiatrists (KN and TA) reviewed the results of the cognitive assessments and confirmed that participants did not meet the criteria for dementia based on those of the National Institute on Aging and Alzheimer's Association and Alzheimer disease Neuroimaging Initiative 2 [40].

### Speech Data Collection

We simulated conversations with a voice assistant on modern smart speakers and smartphones and collected the speech data while performing 3 typical task scenarios: information retrieval (asking for tomorrow’s weather), shopping online (booking a movie ticket), and personal schedule management (creating a calendar event). The tasks began with a simple scenario and then advanced to the more complicated ones. Each task started with an initiating question from the system (“what can I help you with?”), with follow-up questions that asked for detailed information related to the task. The follow-up questions were presented in a fixed order. The questions consisted of four categories—open-ended, to which participants responded with a free-form sentence (Multimedia Appendix 1: Table S1); multiple choice, to which participants responded by choosing one of the options stated in the question; prepared input, to which participants responded with information (eg, passcode) specified by the experimenter; and confirmation, to which participants responded by accepting or rejecting a statement made by the system. The system presented at least 22 questions in total to each participant for the 3 tasks.

To simulate conversations, we took a Wizard-of-Oz [41] approach, in which the participants were told that they were talking with a computer system, though in fact the interaction was mediated by an experimenter (ie, the wizard). We chose this approach so that we could avoid uncertain factors such as errors in automatic speech recognition. During the tasks, the experimenter made the system present a question. After the participant responded, the experimenter prompted the system to move onto the next question if the response contained the necessary information corresponding to the question; otherwise, they would repeat the same question. Each open-ended, multiple choice, and prepared input question presented by the system was scripted in advance and the same for all participants. For confirmation questions, we prepared several variations for each question and the experimenter chose one, depending on the participant’s previous response. For example, the experimenter chose “you are purchasing one ticket, is it OK?” or “you are purchasing two tickets, is it OK?” to have the participant confirm the number of tickets to book.

**Figure 1:**
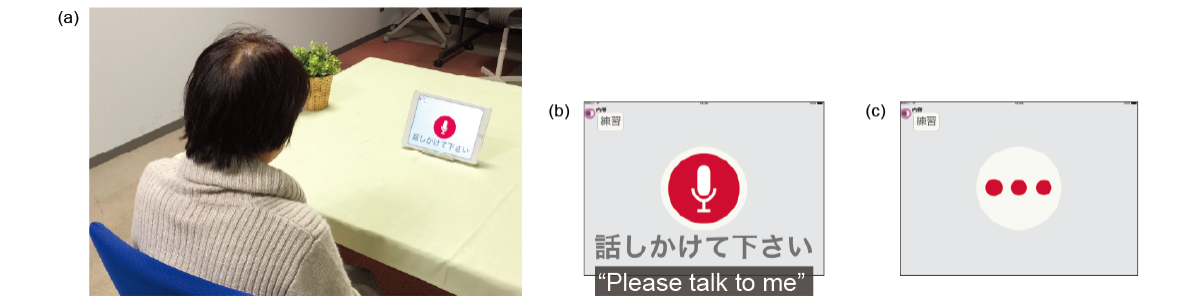
Overview of experimental setup: (a) setup for collecting speech data, (b) screen showing participant's turn, and (c) screen showing the tablet's turn.

The interface for speech data collection was implemented as a tablet-based app on an Apple iPad Air 2. In the experiment, participants sat down in front of the tablet and talked with the system (Figure 1a). During the tasks, the tablet showed a screen indicating whether it was speaking (Figure 1b) or listening (Figure 1c). The experimenter sat behind the participant and operated the system by using a separate interface hidden from the participants. Speech data were recorded in raw format with a sampling rate of 44.1 kHz through the embedded microphone in the tablet. Each experimental session took approximately 30 minutes per participant, including instructions and wrap-up. Additional details about our apparatus and procedure have been previously published [37].

### Data Analysis

From each participant’s speech data, we automatically extracted 84 paralinguistic speech features used in previous studies on inferring cognitive impairments and detecting early signs of Alzheimer disease [19,20,23,27-29,31,42,43]. They consisted of 56 acoustic features and 28 prosodic features.

The acoustic features consisted of features related to mel-frequency cepstral coefficients (MFCCs), jitter, and shimmer. We used the mean and first-order derivatives of the first 12 MFCCs, which represent the short-term power spectrum of the speech signal. Jitter and shimmer features measure cycle-to-cycle variations of fundamental frequency and amplitude [44]. Prosodic features included speech rate, pitch variability, phonation time, number of phonemes needed for completing tasks, response time, total pause duration, and proportion of long pauses (pauses >0.8 seconds). Both acoustic and prosodic features were extracted from each task’s speech data separately. We used Python (version 3.8) audio-processing libraries (librosa, version 0.8.0 [45]; Signal_Analysis, version 0.1.26 [46]).

Statistical analyses were performed using Statistics and Machine Learning Toolbox (version 11.1) for MATLAB (version R2017a, The MathWorks Inc) environment. To assess the differences in each variable between participants with and without accident or near-accident experiences, we used 2-sided Mann-Whitney tests for continuous data and chi-square tests for categorical data. We did not correct for multiple comparisons, and *P* values <.05 were considered significant.

The prediction models for differentiating individuals with and without accident or near-accident experiences were built using multiple types of binary classifiers with automatic sequential forward selection of features. Model performance was evaluated with both leave-one-subject-out cross validation and 100 iterations of 10-fold cross-validation methods. The classifiers included *k*-nearest neighbors [47], random forest [48] and support vector machine [49]. The parameters that we studied were as follows: the number of neighbors for the *k*-nearest neighbors; the number and the maximum depth of trees for random forest; kernel functions, penalty parameter, and the parameter associated with the width of the radial basis function kernel for the support vector machine. We performed an exhaustive grid search to determine these parameters. The algorithms were implemented using the Python scikit-learn package (version 0.23.2).

## Results

For speech data collection (the 30-minute sessions), we obtained an average of 23.8 responses within 100.2 seconds (SD 28.6) from each participant. The average response duration of each task scenario ranged from 17.4 to 59.6 seconds (mean 33.41, SD 9.5). The average duration of a single response for each participant ranged from 1.1 to 7.6 seconds (mean 4.2, SD 1.1). At follow-up, 26 of the 60 participants (43.3%) reported car accident or near-accident experiences within the previous year. Of those, 23 participants reported a near-accident experience, 2 reported accidents, and 1 reported both. The near-accidents consisted of near-misses with a car or pedestrian resulting in a sense of fear and anxiety (eg, from failure to notice a crossing pedestrian), errors in operation (eg, stepping on the accelerator instead of the brake), and unintentional violations (eg, entering the opposite lane).

**Figure 2:**
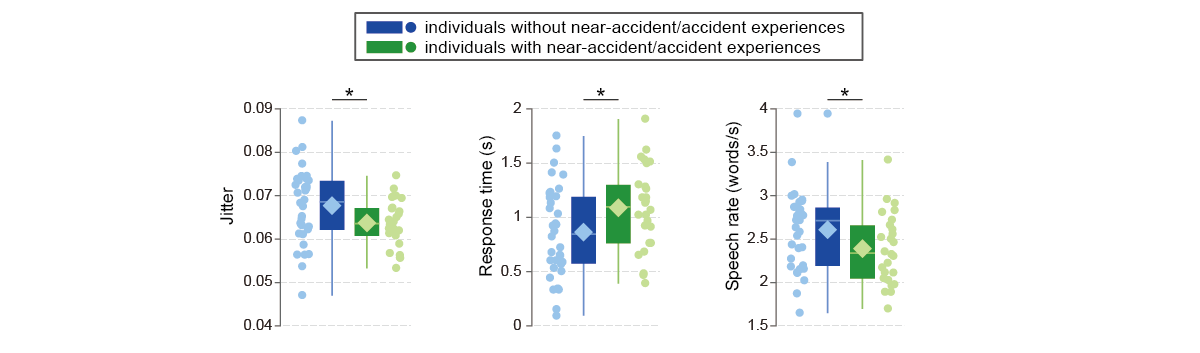
Box plots (line and diamond represent median and mean, respectively) for speech features with significant differences between individuals with and without accident or near-accident experiences—jitter: *P*=.034; response time: *P*=.040; speech rate: *P*=.048.

In comparisons between individuals with and without accident or near-accident experiences, there were no significant differences in any cognitive assessment variables (age: *P*=.45; education year: *P*=.93; sex: *P*=.53; Mini-Mental State Examination: *P*=.28; immediate and delayed recall of the logical memory-story A of the Wechsler memory scale-revised: *P*=.43, *P*=.74; the Frontal Assessment Battery: *P*=.45; the trail making test-part A and B: *P*=.72, *P*=.71; the clock drawing test: *P*=.36; Geriatric Depression Scale: *P*=.62; severity scores for atrophy in medial temporal structures: *P*=.86; Table 1); however, we found 10 speech features with significant differences—ΔMFCC_1_: *P*=.005, ΔMFCC_4_: *P*=.043, ΔMFCC_5_: *P*=.011, ΔMFCC_7_: *P*=.035, ΔMFCC_12_: *P*=.023; jitter: *P*=.034; response time: *P*=.040; proportion of long pauses: *P*=.044; speech rate: *P*=.048; and number of phonemes needed for completing tasks: *P*=.049 (Figure 2; Multimedia Appendix 1: Table S2). Those with accident or near-accident experiences showed decreased speech rate and jitter as well as increased response time and long pauses. These speech features were reported in previous studies as significant indicators of changes in cognitive function, and the trends in their changes were consistent with those observed in individuals with cognitive impairments and patients with Alzheimer disease and mild cognitive impairment (for speech rate [23,27,31]; for jitter [42,43]; for response time [20,27]; for proportion of long pause [27,28]).

**Figure 3:**
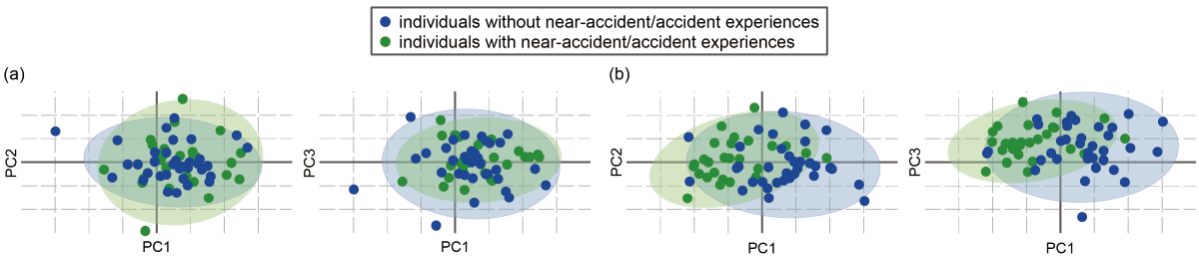
Principal component analysis plots using (a) cognitive assessment variables and (b) speech features, with confidence interval ellipsoid set to 0.95. PC: principal component.

To visualize whether the variance seen among a variable set is capable of discriminating between individuals with and without potential future accident or near-accident experiences, we performed principal component analysis on 2 variable sets: the 12 cognitive assessment variables and 10 speech features (Figure 3). The cognitive assessment variable set had little capability to differentiate the groups; there was considerable overlap and no clear separation. In contrast, the speech variable set enabled some separation of the groups.

**Figure 4:**
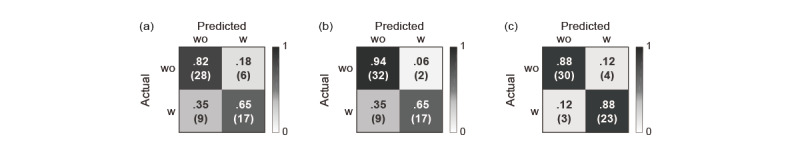
Confusion matrixes for predicting future accident risks of older drivers obtained using leave-one-subject-out cross-validation for models with (a) cognitive assessment variables, (b) speech features, and (c) cognitive assessment variables and speech features combined. The number in parentheses indicates the number of participants. wo: without; w: with.

Input variables for the classification models were either or both the 12 cognitive assessment variables and 10 speech features. When model performance was evaluated with leave-one-subject-out cross-validation, with only the cognitive assessment variables, we obtained 75.0% accuracy (65.4% sensitivity, 82.4% specificity, and 69.4% F1 score; Figure 4a), with only the speech features, the model accuracy increased to 81.7% accuracy (65.4% sensitivity, 94.1% specificity, and 75.6% F1 score; Figure 4b), and with speech features and cognitive assessment variables combined, performance improved further (88.3% accuracy, 88.5% sensitivity, 88.2% specificity, and 86.8% F1 score; Figure 4c). When we evaluated the model using 10-fold cross validation, the results showed similar trends (Multimedia Appendix 1: Table S3): the model using the cognitive assessment variables achieved 75.5% accuracy (95% CI 75.1-75.9), the model using speech features achieved 80.1% accuracy (95% CI 79.7-80.5), and the model using both types of features achieved 85.5% accuracy (95% CI 85.1-85.9).

## Discussion

### Principal Results

In light of the increasing demand for preventing car accidents involving older adults, we investigated the possibility that future accident risk related to cognitive impairments could be automatically predicted with passive unobtrusive monitoring. To this end, we focused on speech data because many previous studies have succeeded in quantifying and detecting cognitive impairments from speech data [19,20,23,27-29,31,42,43], speech data are becoming more accessible, and voice-based interaction systems such as voice assistants are becoming more popular [32-34].

The statistical analysis showed that the speech data collected during typical tasks on smart speakers and smartphones had statistically discernible speech features between older drivers with and without accident or near-accident experiences. These speech features indicated that older drivers with these experiences tended to show decreased speech rate and jitter as well as increased response time and long pauses. These changes in speech features were reported as statistically significant signatures for cognitive impairments by previous studies on patients with Alzheimer disease and mild cognitive impairment [19,20,23,27-29,31,42,43]. The results suggest that speech features could capture subtle impairments of cognitive function in older drivers. On the other hand, we found no differences in any cognitive assessment variables, but this could be explained by the criteria for driving risks that differed from those in previous studies [7,10,12,50-53]. While previous studies compared older drivers with and without car-accident experiences regardless of having near-car-accident experiences and reported significant differences in cognitive assessment scores between them [7,10,12,50-53], we focused on both accident and near-accident experiences, and the majority of the high-risk group in our study were individuals with near-accident experiences but without actual car accidents. Speech data and cognitive assessment results suggest that eliciting discernible changes relevant to future near-accident experiences may require cognitive assessment for subtle impairments, such as, test batteries used for screening preclinical Alzheimer disease [54,55]. Even so, if speech data during interactions with voice assistants can be used for predicting future accident risk, it would greatly increase the accessibility of early screening with a relatively low burden.

The classification model using speech features achieved 81.7% accuracy, which is 6.7% higher than that using cognitive assessment data, and models achieved up to 88.3% accuracy with both combined. Dimensional reduction and visualization using principal component analysis, an unsupervised method, showed that the feature space with speech data was better able to separate those with and without accident or near-accident experiences than the feature space with cognitive assessment variables. These results and those of the statistical analysis indicate that speech data during typical tasks with voice assistants could have comparable (or possibly more) information for predicting future accident risks of older drivers compared with the standard cognitive assessments.

Our results show paralinguistic speech characteristics were useful for predicting future accident risks of older drivers. Previous user-interface studies reported that voice input was effective and was preferable as an input modality for older adults [56-58], while other studies reported that the performance of automatic speech recognition tended to be worse in older adults than in other age groups [59,60]. From this perspective, our results suggest that models for predicting future accident risks of older drivers can be made robust against errors of automatic speech recognition by exploiting paralinguistic features.

Our results highlight the possibility that cognitive impairments related to future car accident risks could be detected using speech data collected in everyday life. Assistive and automated driving systems are promising technologies that may help older adults with cognitive challenges to safely continue driving [61]. Recent studies suggested the importance of individual differences in cognitive abilities for assistive and automated driving technologies for older adults [62,63] because literature has suggested that cognitive abilities affect both performance with automated technology and perceptions of automation (ie, trust) [64,65]. Hence, our approach to detect cognitive impairments associated with driving risks might provide useful information for the personalization of assistive and automated driving systems based on the cognitive abilities of older adults.

### Limitations

Our work had several limitations. First, we collected speech data in a lab setting. The controlled setting might affect the way people interact with a voice assistant. In future work, data collection in free-living situations using voice assistants would be needed along with additional interaction scenarios. Second, the sample size was limited. In spite of this limitation, our statistical analysis of speech features showed consistent trends indicating subtle cognitive impairments in older adults with future accident or near-accident experiences, and the prediction performance (to predict independent future accidents) using speech features was as high as 88%, even when the classifier was trained on a subsample. From these perspectives, we believe that our results can be confirmed by future studies. Third, our definition of future car accident risks was based on self-reports of accident and near-accident experiences. In future work, we need to consider obtaining more objective measures for accident risks by combining self-reports with on-road driving assessments, informant reports, or drive recorder videos.

### Conclusion

Given the increasing demand for car accident prevention involving older adults, we explored the possibility of predicting future accident risks associated with cognitive impairments by using behavioral data that can be collected in everyday life. To this end, we focused on speech data collected during interactions with voice assistants in smart speakers and smartphones and investigated the associations with future accident risks by following up with older drivers. We found that (1) older drivers with accident or near-accident experiences had statistically discernible changes in speech features, implying cognitive impairments, and (2) the machine learning model using speech features could predict future accident or near-accident experiences with up to 88.3% accuracy. Although further studies with speech data collected in everyday life and objective data for near-accidents are needed, our study provides the first empirical results suggesting that speech data during interactions with voice assistants in smart speakers and smartphones could help predict future accident risks of older drivers by capturing subtle impairments in cognitive function. We believe that our results can be used in future efforts toward preventing driving accidents of older adults through continuous passive unobtrusive monitoring.

## Appendix

Multimedia Appendix 1 Supplementary tables.

## Abbreviations

MFCC: mel-frequency cepstral coefficients
MRI: magnetic resonance imaging
