# Supplementary material for: Using Speech Data From Interactions With a Voice Assistant to Predict the Risk of Future Accidents for Older Drivers: Prospective Cohort Study
Source: J Med Internet Res. 2021 Apr 8;23(4):e27667. doi: 10.2196/27667 (PMC8063093; doi:10.2196/27667)
Supplement: Multimedia Appendix 1 [file jmir_v23i4e27667_app1.pdf]

Table S1. Examples of open-ended conversations (translated from Japanese) and topics of follow-up questions for each task scenario. Each conversation consists of questions by the system (*S*) and responses by the participant (*P*). The example for the "information retrieval" shows a complete conversation. The examples for "shopping online" and "personal schedule management" show the beginning of a longer conversation, which is followed by follow-up questions.

Task	Example Conversation (Beginning of Task Scenario)	Follow-up Question Topics
**Information retrieval** (Asking for tomorrow's weather)	S: What can I help you with?<br>P: What will the weather be like tomorrow in this city?<br>S: It will be rainy tomorrow in this city.<br>P: Thank you.	(No follow-up questions)
**Shopping online** (Booking a movie ticket)	S: What can I help you with?<br>P: I want to book a movie ticket.<br>S: What kind of movie would you like to watch?<br>P: Ummm… I'd like a… (pause) comedy film.	• Choose a movie to watch<br>• Choose a date and time<br>• Specify the number of tickets to book<br>• Enter the passcode for payment
**Personal schedule management** (Creating a calendar event)	S: What can I help you with?<br>P: Please add a calendar event to watch a movie.<br>S: Sorry, I couldn't understand what you said. Please try again.<br>P: Well, … could you… ummm… add an event please?	• Choose a date and time<br>• Enter the title of the event<br>• Specify the time for an alarm

Table S2. Characteristics by speech features during interactions with a voice assistant in simulating scenarios on smart speakers and smartphones. ΔMFCC represents the first derivative of MFCC.

	Mean (SD)		
**Speech feature**	**Individuals without accident/near-accident experiences (N=34)**	**Individuals with accident/near-accident experiences (N=26)**	***P* Value**
$\Delta MFCC_1$	-0.41 (5.68)	2.92 (6.67)	.005
$\Delta MFCC_5$	1.22 (1.14)	0.52 (1.10)	.011
$\Delta MFCC_{12}$	-0.19 (0.71)	-0.65 (0.80)	.023
Jitter	0.07 (0.01)	0.06 (0.01)	.034
$\Delta MFCC_7$	0.31 (1.15)	-0.22 (0.84)	.035
Response time, sec	0.86 (0.43)	1.08 (0.39)	.040
$\Delta MFCC_4$	0.28 (1.22)	-0.37 (1.06)	.043
Proportion of long pauses	0.31 (0.20)	0.42 (0.19)	.044
Speech rate, words/sec	2.60 (0.45)	2.39 (0.41)	.048
Number of phonemes needed for completing tasks	26.65 (11.18)	29.04 (7.56)	.049

Table S3. Prediction performance of each model using three different types of input resulting from 100 iterations of 10-fold cross validation. Each value indicates the average value across 100 iterations with a 95% confidence interval.

Input variables	Accuracy	Sensitivity	Specificity	F1 score
Cognitive assessment variables	75.5 [75.1, 75.9]	59.3 [58.5, 60.1]	87.8 [87.4, 88.3]	67.6 [67.0, 68.3]
Speech features	80.1 [79.7, 80.5]	65.7 [65.1, 66.4]	91.1 [90.5, 91.7]	74.1 [73.6, 74.7]
Cognitive assessment variables + speech features	85.5 [85.1, 85.9]	75.8 [75.3, 76.3]	93.0 [92.4, 93.5]	82.0 [81.5, 82.4]
